# Sulfatase‐mediated manipulation of the astrocyte‐Schwann cell interface

**DOI:** 10.1002/glia.23047

**Published:** 2016-08-18

**Authors:** Paul O'Neill, Susan L. Lindsay, Andreea Pantiru, Scott E. Guimond, Nitish Fagoe, Joost Verhaagen, Jeremy E. Turnbull, John S. Riddell, Susan C. Barnett

**Affiliations:** ^1^ Institute of Infection, Inflammation and Immunity, 120 University Place, University of GlasgowGlasgowG12 8TAUnited Kingdom; ^2^ Department of Biochemistry, Centre for GlycobiologyInstitute of Integrative Biology, University of LiverpoolLiverpoolL69 7ZBUnited Kingdom; ^3^ Laboratory for NeuroregenerationNetherlands Institute for NeuroscienceMeibergdreef 47AmsterdamBA1105the Netherlands; ^4^ Institute of Neuroscience and Psychology, West Medical Building, University of GlasgowGlasgowG12 8QQUnited Kingdom

**Keywords:** astrocytes, olfactory ensheathing cells, Schwann cells, sulfatase

## Abstract

Schwann cell (SC) transplantation following spinal cord injury (SCI) may have therapeutic potential. Functional recovery is limited however, due to poor SC interactions with host astrocytes and the induction of astrogliosis. Olfactory ensheathing cells (OECs) are closely related to SCs, but intermix more readily with astrocytes in culture and induce less astrogliosis. We previously demonstrated that OECs express higher levels of sulfatases, enzymes that remove 6‐O‐sulfate groups from heparan sulphate proteoglycans, than SCs and that RNAi knockdown of sulfatase prevented OEC‐astrocyte mixing in vitro. As human OECs are difficult to culture in large numbers we have genetically engineered SCs using lentiviral vectors to express sulfatase 1 and 2 (SC‐S1S2) and assessed their ability to interact with astrocytes. We demonstrate that SC‐S1S2s have increased integrin‐dependent motility in the presence of astrocytes via modulation of NRG and FGF receptor‐linked PI3K/AKT intracellular signaling and do not form boundaries with astrocytes in culture. SC‐astrocyte mixing is dependent on local NRG concentration and we propose that sulfatase enzymes influence the bioavailability of NRG ligand and thus influence SC behavior. We further demonstrate that injection of sulfatase expressing SCs into spinal cord white matter results in less glial reactivity than control SC injections comparable to that of OEC injections. Our data indicate that sulfatase‐mediated modification of the extracellular matrix can influence glial interactions with astrocytes, and that SCs engineered to express sulfatase may be more OEC‐like in character. This approach may be beneficial for cell transplant‐mediated spinal cord repair. GLIA 2016 GLIA 2017;65:19–33

## Introduction

Schwann cells (SCs) are an attractive candidate for cell‐transplantation following spinal cord injury (SCI). They effectively fill cavities, reduce tissue loss and promote regeneration and remyelination of CNS axons (Blakemore, 1977; Duncan et al., 1981). Human SCs can be efficiently cultured in vitro, meaning that large numbers of cells are available for therapeutic use (Rutkowski et al., 1995). Used in isolation however, SCs do not result in significant improvements in functional outcome in experimental models of SCI (Martin et al., 1996; Pearse et al., 2007). The limited functional success of SC transplantation is due in part to limited SC migration and integration within the CNS and their inability to survive and myelinated axons in astrocyte‐rich regions (Blakemore et al., 1986; Iwashita and Blakemore, 2000; Iwashita et al., 2000). Astrocytes encountering SCs in the spinal cord upregulate GFAP and eventually isolate the SCs from the rest of the CNS (Shields et al., 2000).

This phenomenon can be mimicked in vitro to a certain extent. When cultured SCs and astrocytes are seeded in close proximity to one another they form distinct territories and do not readily intermingle (Ghirnikar and Eng, 1994; Wilby et al., 1999; Lakatos et al., 2000; Santos‐Silva et al., 2007). Olfactory ensheathing cells (OECs) have been proposed as an alternative to SCs for transplantation into spinal injuries (Barnett and Riddell, 2004). OECs are similar to SCs in many ways: they originate developmentally from the neural crest (Barraud et al., 2010), show SC‐like molecular and cellular characteristics (Franceschini and Barnett, 1996; Smith et al., 2001) and can ensheath demyelinated large diameter axons and deposit functional peripheral myelin proteins (Franklin et al., 1996; Imaizumi et al., 1998). The unique tissue niche occupied by OECs, spanning the interface of the CNS and PNS (Raisman, 1985), means that OECs are not restricted in their interactions with astrocytes and can intermix freely (Doucette, 1990; Lakatos et al., 2000).

Despite promising results following OEC transplantation into the injured spinal cord (Ramón‐Cueto et al., 1998; Witheford et al., 2013), they are not ideal for clinical application due to difficulties in culturing large numbers of human OECs (Tabakow et al., 2014). An alternative therapeutic strategy is to modify SCs to be more OEC‐like; specifically, to overcome the normal, inhibitory relationship between SCs and astrocytes. Previously we demonstrated that OECs express higher levels of the extracellular heparan sulfate (HS) 6‐O‐endosulfatases Sulf1 (S1) and Sulf2 (S2) than SCs and that these enzymes modulate OEC‐astrocyte intermingling by altering the sulfation of secreted HS proteoglycans (HSPGs) (Higginson et al., 2012). HSPGs are major, ubiquitous components of the extracellular matrix (ECM) which often act as co‐receptors, modulating signaling in growth factor‐receptor interactions (Gallagher, 2012). A core proteoglycan is linked to multiple HS glycosaminoglycan side chains which can be modified by deacetylation, epimerization, and the addition of N‐ or O‐sulfate groups (Bernfield et al., 1999; Turnbull et al., 2001). Variations in the sulfation profile of HSPGs directly influence the binding affinity of HSPGs to growth factors or their receptors and subsequent signaling. Sulfatases remove 6‐O‐sulphate groups from cell surface HSPGs and modulate the activity of multiple signaling molecules including FGFs, BMPs and Wnts by controlling ligand bioavailability and facilitating ligand‐receptor binding (Ai et al., 2003, 2007; Freeman et al., 2008; Otsuki et al., 2010; Wang et al., 2004). Sulf1 and Sulf2 are unique in that they can specifically hydrolyse glucosamine‐6S groups of the HS chain at the cell surface, and thus postsynthetically edit 6‐0‐sufation patterns (Dhoot et al., 2001; Roop et al., 2016). This reduction in 6‐OH sulfation can promote specific cell signaling pathways e.g. Wnt and GDNF but inhibits others such as tyrosine kinase receptor mediated FGF1, HB‐EGF and HGF pathways. In cancer biology Sulf1 is thought to act as a tumour repressor but Sulf2 as a tumour enhancer (Roop et al., 2016). Moreover it has been shown that the Sulfs can act cooperatively in vivo to modify HS sulfation patterns and regulate development (Lamanna et al., 2006). Thus Sulf1 and Sulf 2 may have different functions in vivo. This view is supported by recent work showing that they differentially regulate HS sulfation during postnatal cerebellum development, resulting in Sulf‐specific interference in signaling pathways (Kalus et al., 2015).

In this study we tested the effects of sulfatase transduction on in vitro and in vivo models of SC‐astrocyte reactivity and demonstrate that sulfatase over‐expression in SCs may positively influence the environment of SC grafts by minimizing local astrocyte reactivity.

## Materials and Methods

### Glial Cell Culture

Primary cells were prepared from Sprague Dawley (SD) or F344‐Tg(UBC‐EGFP)F455Rrrc (RRRC, Rat Resource and Research Center) rat pups according to methods modified from Higginson et al. (2012). Astrocytes were generated from postnatal day one (P1) cortex: tissues were digested with 5 mg/ml collagenase (Sigma‐Aldrich) for 20 min at 37°C, triturated and seeded onto poly‐L‐lysine (PLL: 13 µg/ml Sigma‐Aldrich) coated culture flasks and maintained in DMEM medium containing 10% foetal bovine serum (DMEM‐FBS). Contaminating neural progenitors and fibroblasts were eliminated by overnight treatment with cytosine arabinoside (AraC; 10 µM). SCs were purified from P7 sciatic nerve; approximately 12 nerves were finely chopped with a scalpel blade prior to digestion with 5 mg/ml collagenase (Sigma‐Aldrich) for 20 min, then 2.5 mg/ml trypsin (Sigma‐Aldrich) for 10 min. Digestion was stopped with SD solution containing 0.52 mg/ml Soya bean trypsin inhibitor (Sigma‐Aldrich), 0.04 mg/ml DNAseI (Sigma‐Aldrich) and 3 mg/ml bovine serum albumin (Sigma‐Aldrich). Cells were then plated onto 25cm^2^ PLL‐coated flasks containing Schwann cell media [ScM; DMEM‐FBS supplemented with 0.5 µM forskolin (Sigma‐Aldrich) and 50 ng/ml heregulin β1 (R&D Systems)]. OECs were isolated from P7 olfactory bulbs according to the methods of Barnett et al. (1993). Briefly, olfactory bulbs were, digested with 5 mg/ml collagenase, triturated then plated on PLL coated flasks in DMEM containing 5% FBS, 10% astrocyte conditioned media, 35% DMEM‐BS (defined serum‐free media, Bottenstein and Sato, 1979), 25 mg/ml FGF2 (Peprotech), forskolin (0.5 µM) and heregulin (50 ng/ml). After 7 days in culture, SCs and OECs were purified with magnetic‐beads cross‐linked to p75^NTR^ antibody (Abcam) using an EasySep immunomagnetic positive selection kit (Stem Cell Technologies).

### Generation of Lentiviral Vectors and Schwann Infection

Second generation LV transfer plasmid pRRL‐CMV‐MCS‐WPRE containing a constitutively active human cytomegalovirus (CMV) promoter, a multiple cloning site (MCS) and the woodchuck hepatitis virus post‐transcriptional regulatory element (WPRE) were used to insert the coding sequence of mouse Sulf1 or Sulf2 between the CMV promotor and the WPRE using standard molecular cloning techniques. The Sulf1 and Sulf2 genes were cut from plasmids 13008:pcDNA3.1/myc‐His(‐)‐Msulf2 and 13007: pcDNA3.1/myc‐His(‐)‐Msulf‐1, (Addgene). LV stocks were generated as previously described (Dull et al., 1998; Hendriks et al., 2007; Naldini et al., 1996). Briefly, for each batch of LV, two 15‐cm Petri dishes containing 12.5 × 10^6^ HEK293T in Iscove's modified Dulbecco's medium (IMDM) containing 10% foetal calf serum (FCS), 1% penicillin/streptomycin (P/S) and glutamax (Invitrogen, Carlsbad, CA) were prepared. Cells were maintained at 37°C in a humidified atmosphere of 5% CO^2^. A triple transfection with the LV transfer, packaging (pCMVdeltaR8.74) and envelope (pMD.G.2) plasmid was performed (ratio 3:2:1, total DNA 90 μg per plate) using branched polyethylenimine (Sigma, St Louis, MO, USA). After 14 h, the medium was replaced by Iscove's modified Dulbecco's medium containing 2% FCS, 1% Pen/Strep and glutamax. After 24 h, the medium was harvested, filtered through a 0.22‐μM filter and concentrated by ultracentrifugation at 20 000 r.p.m. for 2.5 h in a SW32Ti rotor (Beckman Coulter BV, Woerden, The Netherlands). Viral pellets were resuspended in PBS pH 7.4, aliquoted and stored at −80°C until further use. Viral vector titers were determined by first determining the amount of viral vector with a p24 ELISA in the LV‐GFP and LV‐SULF1 and LV‐SULF2 stocks. Subsequently the titer of the LV‐GFP stock in transgene expressing units (TU)/mL was determined by counting GFP expression cells in 24‐well plates transduced with serial dilutions of LV‐GFP. Since the LV‐SULF viruses do not contain a reporter gene the titers of these stocks were calculated by extrapolating the value of the LV‐GFP stock to TU/mL. Titers are 3.59E + 09 TU/mL for LV‐mSulf1 and 3.54E + 09 for LV‐mSulf2. SCs were infected with lentivirus containing full length mouse Sulf1 or Sulf2 sequence. Cells were seeded in 24‐well plates at 1,000 cells/µL and cultured overnight in 500 µL ScM. 1 µL of Sulf1 or Sulf2 lentivirus supernatant (titre: 3.5 x 10^9^ TU/mL) was applied to each well and incubated for 48 h before cells were transferred to 25 cm^2^. PLL‐coated flasks containing ScM. Double infected cells were generated by applying S1 and S2 viruses at the same time.

### PCR

The presence of introduced mouse sulfatase transcripts was confirmed by PCR using standard methods. Briefly, RNA from monocultures of infected SCs was extracted using a Qiagen RNeasy Mini Kit (Qiagen) following manufacturer's instructions. RNA quality and integrity were checked using the Nanodrop 1000 (Thermo Fisher Scientific). Following RNA extraction, cDNA was synthesized from 1 μg of RNA using the QuantiTect Reverse Transcription kit (Qiagen). PCR was performed with 100 ng of cDNA following manufactures instructions (Qiagen). Experiments were performed in triplicate for each sample using the Applied Biosystems 7500 real‐time PCR system. PCR cycle settings were 95°C for 5 min, followed by 40 cycles of 95°C for 10 s, then 60°C for 30 s. GAPDH was used as control. Three independent cell preparations were analyzed. In each case; enzyme function was evaluated by analysis of 6‐O‐sulfation of HS from conditioned SC culture media by HPLC chromatography after BODIPY fluorescent labelling of disaccharides (Guimond et al., 2009; Higginson et al., 2012, Fig. 1D).

**Figure 1:**
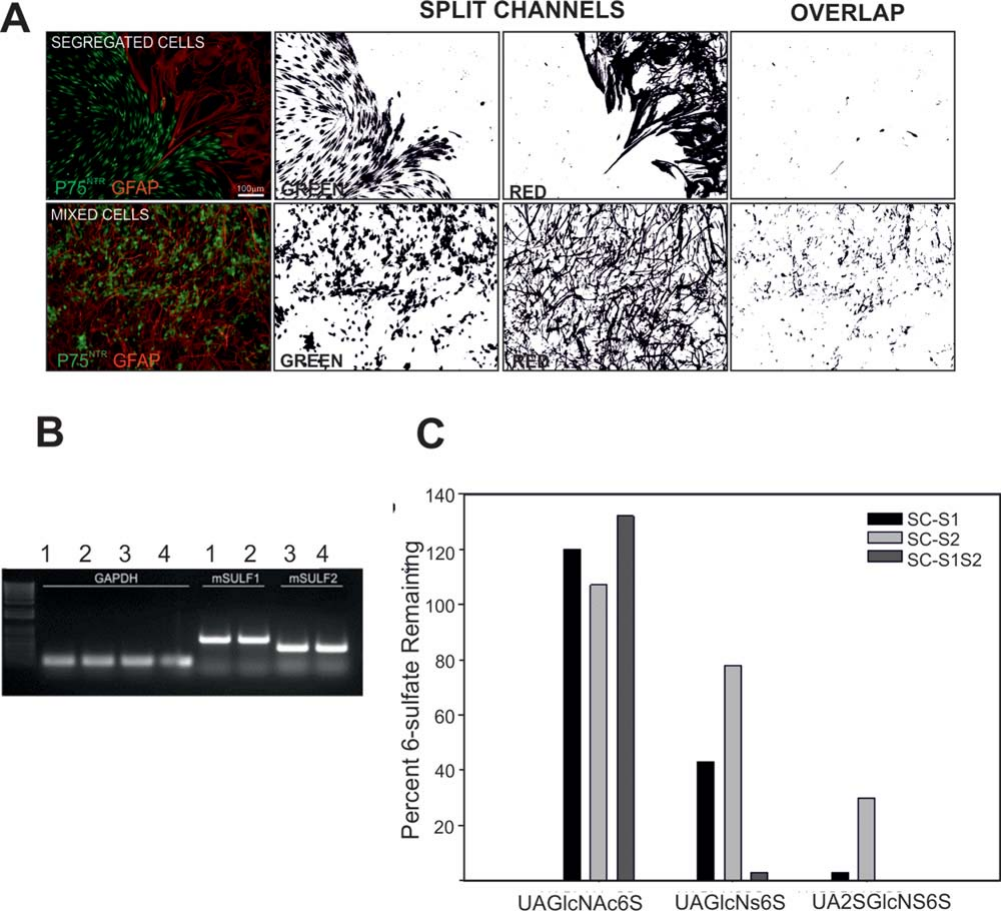
Estimation of cell mixing by measurement of overlapping pixels. **A**: Boundary assays immunostained for p75^NTR^ (green, SCs, or OECs) and GFAP (red, astrocytes) were imaged using a 10X objective. Captured TIFFs were then processed using ImageJ software. Images were split into red and green channels; these were then converted to binary black and white images using the threshold function. The pixel overlap of red and green images was then calculated, resulting in higher overlap measurements for mixed cell populations than for segregated cells. **B**: Image of gel showing the expression of mouse Sulf1 (samples 1 and 2) and Sulf 2 (samples 3 and 4) in lentivirus‐transfected rat SCs. **C**: Heparan sulfate disaccharides containing 6‐sulfate moieties were identified from conditioned medium from SC‐WT, SC‐S1, SC‐S2, and SC‐S1S2 cells. Data are presented as the percentage of the indicated disaccharides present in the transfected cells with reference to the wild type SCs. Each disaccharide consists of one uronic acid and one glucosamine moiety, with structures defined by combinations of: UA, delta4,5‐uronic acid; UA2S, 2‐O‐sulfated delta4,5‐uronic acid; GlcNAc, N‐acetylglucosamine; GlcNAc6S, 6‐O‐sulfated‐N‐acetylglucosamine GlcNS, N‐sulfoglucosamine; GlcNS6S, 6‐O‐sulfated‐N‐sulfoglucosamine. *n* = 4 from 2 technical and 2 biological replicates; SD < 0.1% and are not visible on the figure. [Color figure can be viewed at wileyonlinelibrary.com]

### Boundary Assay

Glial boundary assays were performed based on modifications to the methods of Wilby et al. (1999) and Lakatos et al. (2000). Purified SCs (2000 cells/µL) or OECs (1500 cells/µL) were pipetted onto glass coverslips as a narrow 10 μL cell strip. A second parallel 10 μL strip of astrocytes (1,000 cells/µL) was pipetted immediately adjacent to the first; the meniscus of each cell droplet was then encouraged to touch along the entire length of the cell front by gentle manipulation with a pipette tip. This technique results in SCs or OECs being brought into immediate proximity with astrocytes, but does not result in cell mixing. Cells were allowed to settle for 1 h before the removal of unattached cells by rinsing with 500 µL PBS and incubation with 500 µL of ScM. Within 2 days, distinct SC or OEC, and astrocyte territories were visible; the breakdown of the boundaries dividing these cellular territories forms the basis of our assay. Boundary assays were fixed with 4% PFA after 1 week in culture. This assay is distinct from our previous “confrontation” assays in several ways (Lakatos et al., 2000). In our original confrontation assay cells were placed in strips opposing each other and allowed to grow towards each other for 7 − 10 days in DMEM containing 10% FBS (Lakatos et al., 2000). In the modified assay the meeting of the cells is immediate, and the cultures were carried out in ScM (i.e., DMEM‐FBS containing 50 ng/mL heregulin β1and 0.5 µM forskolin) to enhance SC viability. We found SCs had a greater variability in viability when the SCs were cultured in DMEM‐10% alone. Moreover these modifications allows for a more moderate throughput since timing for contact is faster. Lastly an objective macro was generated which could automatically measure green/red pixel overlap to assess cells crossing boundaries.

### Treatment of Boundary Assays

The following reagents were added to media: 25 ng/ml FGF2 (Peprotech); 25 ng/mL FGF9 (Peprotech); 5 µM GSK1059615 (phosphatidylinositol 3‐kinase (PI3K) inhibitor, Sigma‐Aldrich); 5 µM SU5402 (FGF‐receptor blocker, Sigma‐Aldrich); 10 µM Wortmannin (PI3K inhibitor, Sigma‐Aldrich); 10 µM PD98059 (MAPK inhibitor Sigma‐Aldrich); 10 µM AKT1/2 kinase inhibitor (AKT inhibitor, Sigma‐Aldrich); 1 µM XAV939 (Wnt/β‐catenin inhibitor, Sigma‐Aldrich); 0.5 µM DMH1 (BMP inhibitor, Sigma‐Aldrich); 0.8 ng/mL EGFR/ErbB‐2/ErbB‐4 inhibitor (NRG/HRG blocker, Calbiochem); 50 − 250 mg/mL Heregulin β1 (also known as neuregulin β1; PeproTech, UK). All treatments were applied immediately after boundary assays were set‐up and were administered every 2 days during routine culture feeding for 1 week.

### Immunocytochemistry

Cultures were fixed in 4% paraformaldehyde (PFA) for 10 min at room temperature (RT), then rinsed with PBS. Primary antibodies (P75^NTR^ IgG1, 1:1000, Abcam; GFAP anti‐rabbit, 1:1000, DAKO) were diluted in 0.1% Triton‐PBS (PBST) containing 5% FBS and applied overnight at 4°C. Samples were washed three times in PBST before incubation with fluorescently conjugated secondaries (Alexa‐488, Alexa‐555, Life Technologies) for 1 h at RT. After three washes with PBST, coverslips were mounted using an aqueous mounting solution containing DAPI (Fluoroshield™, Sigma‐Aldrich). Specimens were viewed under epifluorescence with an Olympus BX51 microscope; images were captured using a QImaging EXi Aqua camera and Image‐Pro software.

### Measurement of Overlap for 2 Cell Populations in Boundary Assays

In order to standardize images and allow meaningful comparisons to be made, each field of view was required to comprise of approximately equal numbers of the cell types under investigation (i.e., at the position of the initial boundary), and contain no empty acellular spaces or patches of dead cells. All images were captured using a 10x objective and exposure times were standardized for each experiment. Each TIFF file was imported into ImageJ software where the image was split into individual color channels. Next, the threshold of the red and green color channels, representing GFAP and p75^NTR^ immunostaining respectively, was adjusted to generate a black and white image; this was then inverted using the “Make Binary” function, resulting in a black signal on a white background (see Fig. 1A). Care was taken during this procedure that the final binary image faithfully represented the initial staining. The auto‐threshold function using the “Default” setting reliably produced good results from our images. Using “Image Calculator” function in ImageJ, we generated a third image representing the regions of overlap between the binary green and red images. This was achieved using the “AND” operation, resulting in positive output only in positions where black pixels are present in both green and red binary input files. The number of overlapping pixels was established by counting total black pixel number in the new green‐red overlap image using the Histogram function (total number of pixels with value 255). This procedure was repeated for each field of view from all coverslips. A minimum of 4 images was taken from each stained coverslip, and each experiment consisted of at least 3 independent coverslips. This data was used to determine an average measurement of cell overlap for each experimental condition; each experiment was repeated at least three times with different cells (see figure legends for individual n‐values). Statistical significance was calculated using one‐way ANOVA with Dunnett's multiple comparison test.

### Cell Injections into the Spinal Cord

Twenty‐four adult male Sprague Dawley rats (200–250g; Harlan Laboratories, Loughborough, UK) were used in the study. Animals were housed under a 12 h light/dark cycle with ad libitum access to food and water. All experimental procedures were performed in accordance with the United Kingdom Animals (Scientific Procedures) Act 1986. Animals were anesthetized with isoflurane and placed in a head holder (Kopf, USA) on a homeothermic blanket. The T2 dorsal process was exposed and clamped to stretch and stabilize the cervical vertebrae and a laminectomy performed to expose the spinal cord from the C3 to C7 segmental level. Cells (SCs or OECs) prepared from RRRC GFP‐rats and purified as above were injected into the exposed spinal cord using a micropipette mounted in a stereotaxic manipulator with a Microdrive. Immediately before transplantation, cells were detached from flasks with 0.25% trypsin‐EDTA (Sigma‐Aldrich), counted using a haemocytometer and resuspended at a concentration of 300,000 cells/µl in DMEM‐FBS containing DNAseI (10 µg/ml). Cells were kept on ice until being drawn into a pulled glass pipette (GC 100T‐100 capillaries, Harvard Apparatus), with a bevelled tip of approximately 60‐70 µm diameter. For dorsal column (white matter) injections, pipettes were inserted vertically at points 100 µm lateral from the midline, and to a depth of 600 µm. Cells were slowly injected by application of brief (40 ms) pressure pulses (Picoinjector, WPI, Sarasota FL), as the pipette tip was withdrawn from 600 to 300 µm. For dorsal horn (gray matter) injections, pipettes were inserted ∼550 µm lateral from the midline and cells were injected as the needle was withdrawn from 800 − 500 µm depth. Each animal received 5 or 6 separate injections with a rostro‐caudal separation of ∼2 mm. A 10‐0 ethicon suture was placed in the dura at a known distance from the most rostral injection site to facilitate identification of injections sites when removing the spinal cord for histological processing. The wound was closed and animals recovered in warmed cabinets overnight. Animals received analgesia (buprenorphine, 0.05 mg/kg and carprofen, 5 mg/kg, s.c., at induction of anaesthesia and the morning after surgery).

### Free Floating Immunostaining of Tissue Sections

Ten days following SC injection into uninjured spinal cords, animals were injected intraperitoneally with 200 mg/mL sodium pentobarbital (Euthatal, Vericore, UK) to induce deep anaesthesia, then perfused though the left ventricle with ∼50 mL mammalian Ringer's solution (containing 0.1% lidocaine), followed by 1,000 mL of 4% PFA in PBS. The cervical region of spinal cord was carefully removed and post‐fixed overnight in 4% PFA containing 30% sucrose at 4°C. 60 µm transverse sections were cut using a cryostat, then incubated in 50% ethanol for 30 min at RT in 7 ml glass bottles. After washing in PBS, sections were incubated for 72 h with primary antibodies (GFAP, 1:1,000, DAKO; nestin, 1:500, Chemicon; anti‐GFP, 1:1,000, Abcam) diluted in PBS containing 0.3% Triton‐X (PBST). Samples were then washed with 3 changes of PBS, then incubated for 4 h with appropriate fluorescent secondary antibodies (IgG1, anti‐rabbit, anti‐sheep or chicken respectively, Jackson ImmunoResearch Lab) diluted in PBST. Finally sections were washed 3 times in PBS, mounted onto glass slides with VectaShield (Vector Laboratories), and stored at −20°C before imaging with a Zeiss LSM 710 confocal microscope.

### Analysis of Reactivity of Cell Injection Sites

Reactivity of surrounding spinal cord tissue was estimated by immunostaining for GFAP and nestin proteins. Both are upregulated following spinal cord damage, although neither is limited uniquely to the injury site. Overlap of GFAP and nestin, however, is entirely restricted to reactive astrocytes surrounding the injury site and we therefore based our reactivity measurements on this parameter. Maximum intensity projections derived from 2 x 2 confocal tiles (40X objective) surrounding each injection site were saved as TIFF files and imported into ImageJ. From this, imageJ was used to split channels and threshold GFAP and nestin signals (Fig. 7A). Overlap of GFAP/nestin immunoreactivity was recorded for each tissue section; a minimum of 3 tissue sections was imaged from each individual injection site. At least 4 experimental animals were evaluated per experiment; each animal received 5 or 6 separate spinal cord injections. Statistical significance was calculated using one‐way ANOVA with Dunnett's multiple comparison test. Transplanted cells were visualized using anti‐GFP.

## Results

### Sulf Transduction Modulates Schwann Cell Motility in Vitro

The interaction of transplanted glia and endogenous astrocytes can be modelled using in vitro boundary assays (Wilby et al., 1999). These “boundaries” are a proxy for the SC‐astrocyte reactive response in vivo. For analysis, an ImageJ macro was designed that examined the extent of SC/OEC and cortical astrocyte mixing by calculating the degree of overlap of GFAP and p75^NTR^ immunolabeling across the boundary region (Fig. 1A).

**Figure 2:**
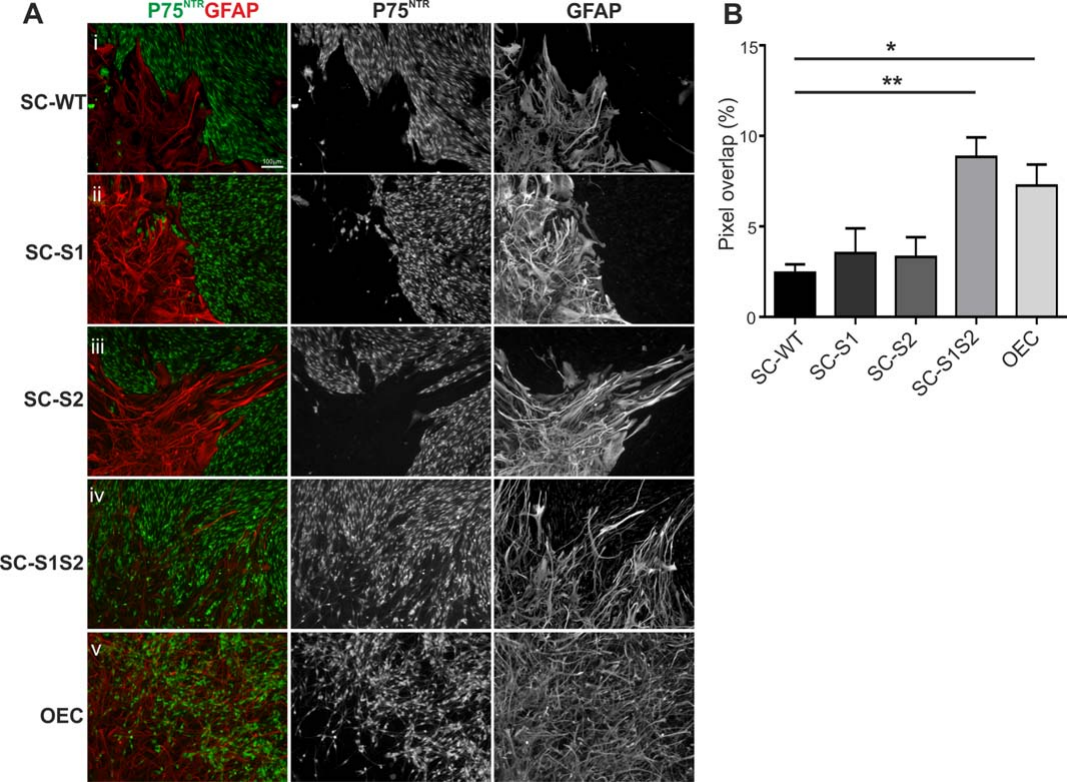
Sulfatase transduction increases motility of Schwann cells in astrocytic environments. **A**: Cell mixing was measured in SC‐WT‐astrocyte or OEC‐astrocyte boundary assays immunostained with p75^NTR^ (green) and GFAP (red). (i) Control SC‐WT‐astrocyte experiments resulted in the formation of clear cellular territories with minimal cell mixing. SC‐S1 (ii) or SC‐S2 (iii) did not affect cell behavior: strong boundaries were still formed with astrocytes. SC‐S1S2 however did not form boundaries (iv), cell mixing was significantly greater than controls and was comparable to levels of cell mixing seen in OEC‐astrocyte boundary assays (v). Scale bar represent 100 μm in all images. B) Quantification of cell mixing reveals a significant difference between control SC‐WT and SC‐S1S2 (*n* = 6; *P* = 0.001), and between control SC‐WT and OECs (*n* = 6; *P* = 0.019). [Color figure can be viewed at wileyonlinelibrary.com]

To test the hypothesis that altering the sulfation profile of the local ECM environment may positively affect the outcome of SC interacting with astrocytes, we infected GFP‐labeled SCs with a lentivirus containing full length mouse S1 or S2. The resulting cells were confirmed to express Sulf1 (S1), Sulf2 (S2), or both Sulf1 and Sulf2 (S1S2) using PCR (Fig. 1B), and had elevated sulfatase activity as indicated by analysis of HSPG 6‐O‐sulfation using HPLC. Control nontransduced cells did not show any bands (data not shown). Sulf1 and Sulf2 expression reduced the percentage of di‐ (UA‐GlcNS6S) and tri‐sulfated (UA2S‐GlcNS6S) disaccharides containing 6‐O‐sulfate groups to 40% and 3% of levels in wild type Schwann cells (SC‐WT) (Fig. 1C). The double S1S2 transfected SCs displayed near‐complete elimination of the di and tri‐sulfated saccharides. Interestingly, the monosulfated saccharide UA‐GlcNAc6S was slightly increased in the HS of all transfected cells, indicating that the Sulf enzymes preferentially desulfated the di‐ and tri‐sulfated disaccharides. Another possible but rare 6‐sulfated disaccharide, UA2S‐GlcNAc6S was not detected in the wild type or any of the transfected cells (data not shown). Moreover, although infection of SCs with S1 or S2 lentivirus individually did not affect cell behavior in boundary assays (Fig. 2Ai‐iii), SC‐WT infected with both S1 and S2 resulted in significant mixing of SCs and astrocytes (Fig. 2Aiv) such that mingling was similar to that seen for OECs and astrocytes. Boundary assays using OECs were used as a positive control, and also exhibited significantly higher levels of mixing with astrocytes than SC‐WT (Fig. 2Av,B). Moreover, proliferation rates of SC‐S1S2 and SC‐WT were found not to be significantly different in medium used in boundary assays (data not shown).

### Mechanisms of Schwann Cell‐Astrocyte Mixing

**Figure 3:**
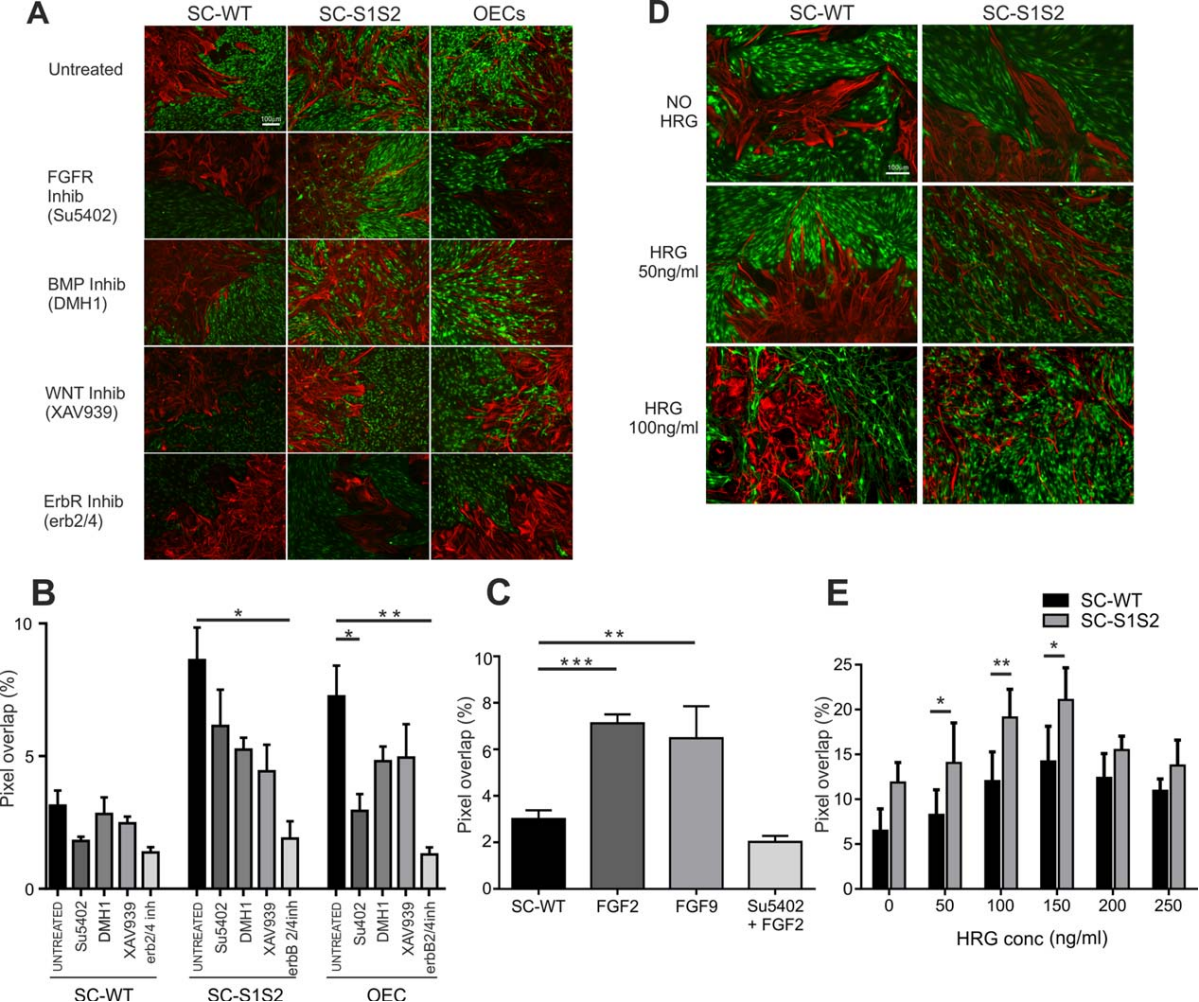
Signaling molecules in boundary formation. **A**: Boundary assays with SC‐WT, SC‐S1S2, and OECs were set up and treated with a range of growth factor inhibitors and immunostained with GFAP (red) and p75^NTR^ (green). The FGF receptor inhibitor SU5402 did not affect the extent of cell mixing in control or SC‐S1S2 experiments, but did block cell mixing in OEC‐astrocyte cultures. BMP and Wnt inhibition did not significantly affect cell mixing in all cases tested. Blockade of erbB receptor signaling resulted in the formation of strong boundaries in SC‐WT, SC‐S1S2, and OECs with astrocytes. **B**: Quantification of cell mixing illustrated in (A). ErbB receptor inhibitor significantly blocked cell mixing in SC‐S1S2‐astrocyte cultures (*n* = 3; *P* = 0.01). OEC‐astrocyte mixing was inhibited by FGF‐receptor inhibitor (*n* = 5; *P* = 0.017) and by erbB inhibitor (*n* = 4; *P* = 0.002). **C**: Quantification of SC‐astrocyte mixing after FGF treatments. FGF2 significantly elevated SC‐astrocyte intermingling (*n* = 4; *P* = 0.0002), as did FGF9 (*n* = 3; *P* = 0.003). This mixing was blocked by the addition of the FGF blocker SU5402. **D**: Boundary assays immunostained with GFAP (red) and p75^NTR^ (green) in the presence of varying concentrations of HRG. **E**: Increased concentrations of HRG promote cell mixing. Normal boundary assay concentrations of HRG (50 ng/mL) and HRG concentrations of 100 ng/mL and 150 ng/mL resulted in significantly more SC‐S1S2‐astrocyte mixing (*n* = 6), than in control SC‐WT‐astrocyte mixing (*n* = 4; *P* = 0.001, *P* = 0.005, and *P* = 0.0181, respectively). Increasing concentration of HRG in both SC‐WT and SC‐S1S2 caused a dose‐dependent increase in cell mixing. Scale bar represents 100 μm in all images. [Color figure can be viewed at wileyonlinelibrary.com]

We next applied chemical inhibitors of the FGF, BMP, Wnt, and NRG/HRG receptors to boundary assays to evaluate the importance of each signaling pathway in sulfatase‐mediated SC‐astrocyte mixing. FGF receptor inhibitor SU5402 did not increase cell mixing in SC‐WT or SC‐S1S2‐astrocyte cultures, but showed a trend towards reduced cell mixing (i.e., boundary formation) although this was not statistically significant (Fig. 3A,B). Indeed, treatment of OEC‐astrocyte boundary assays with SU5402 resulted in a significant reduction in cell mixing and the formation of SC‐like boundaries with astrocytes (Fig. 3A,B). These findings implicate FGF activity in glial mixing; this was investigated further by adding recombinant FGF protein to SC‐astrocyte boundary assays. FGF2 or FGF9 application disrupted boundary formation, elevating levels of cell mixing to levels similar to those of SC‐S1S2 or OECs (Fig. 3C). SC‐S1S2‐astrocyte cultures did not show further elevation of cell mixing levels when treated with FGF2 (data not shown), suggesting that the extent of mixing may be at a maximum level. SU5402 effectively prevented the activity of the recombinant FGFs, confirming that the observed effect was FGF‐receptor mediated (Fig. 3C). Chemical inhibitors of the BMP and Wnt signaling pathways were also tested for activity in boundary assays, but had no statistically significant effect (Fig. 3A,B).

As HSPG‐binding is required for neuregulin (NRG also known as heregulin (HRG)) signaling via erbB receptors (Sudhalter et al., 1996), and this interaction is affected by the sulfation profile of HSPG glycosaminoglycan chains (Pankonin et al., 2005), we hypothesised that the increased motility of SC‐S1S2 may be caused by elevated sensitivity to NRG protein. Treatment of boundary assays with an erbB receptor inhibitor significantly reduced cell mixing in all cases (values <5), with SC‐S1S2 and OECs forming strong boundaries comparable to those in control SC‐WT experiments (Fig. 3A,B). Further boundary assays were performed with media containing increasing concentrations of HRG to assess whether media supplementation directly affected SC‐astrocyte mixing. In the absence of HRG, boundaries were formed in SC‐WT‐astrocyte cultures, while SC‐S1S2‐astrocyte cultures showed slightly more mixing although this did not reach statistical significance (Fig. 3D,E). At HRG concentrations typically used to maintain and grow SC in culture (50 ng/ml) SC‐WT form boundaries with astrocytes, while SC‐S1S2‐astrocytes mix (Fig. 3D), and at elevated HRG concentrations (100 ng/ml, 200 ng/ml, 250 ng/ml), both SC‐WT and SC‐S1S2 mix freely with astrocytes (Fig. 3D). Interestingly the SC‐S1S2 response was always greater than SC‐WT. These data suggest that HRG may be a key determinant of SC‐astrocyte mixing and that the sulfation profile of the extracellular environment influences HRG bioavailability and activity.

### PI3 Kinase/AKT Activity Is Critical for Glial Cell Mixing

**Figure 4:**
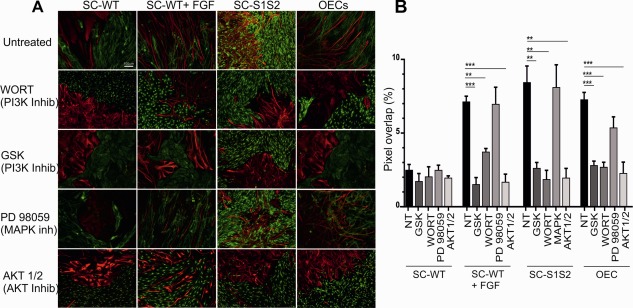
PI3K/AKT‐mediated signaling modulates Schwann cell and OEC interactions with astrocytes. **A**: Treatment of control SC‐WT‐astrocyte cultures with inhibitors of the PI3 kinase signaling cascade (Wortmannin or GSK1059615), did not affect boundary formation. However, PI3K inhibitors blocked cell mixing in SC‐S1S2, SC‐WT (in the presence of FGF2), and OEC‐astrocyte boundary assays, reducing GFAP/p75^NTR^ overlap to levels similar to control SC‐WT experiments. Similarly, inhibition of AKT also blocked cell mixing in SC‐S1S2, SC‐WT (in the presence of FGF2) and OEC‐astrocyte cultures. Chemical inhibition of the MAPK pathway had no effect on cell mixing in any of the cell types examined here. Scale bar represents 100 µm in all images. **B**: Quantification of boundary assays in the presence of the PI3K inhibitor Wortmannin shows a significant reduction in cell mixing compared to controls in FGF‐treated SCs (*n* = 4; *P* = 0.003), SC‐S1S2 (*n* = 3; *P* = 0.008), and OEC (*n* = 5; *P* < 0.0001). The PI3K inhibitor GSK1059615 has similar effects, blocking cell mixing in FGF‐treated SCs (*n* = 3; *P* < 0.0001), SC‐S1S2 (*n* = 4; *P* = 0.009), and OECs (*n* = 7; *P* < 0.0001). Blocking AKT also affected SC‐S1S2 (*n* = 5; *P* = 0.0017), and OECs (*n* = 3; *P* < 0.0001). The MAPK inhibitor PD98059 did not reduce cell mixing in FGF‐treated or SC‐S1S2‐astrocyte assays, but did affect OEC‐astrocyte mixing (*n* = 6; *P* = 0.039). [Color figure can be viewed at wileyonlinelibrary.com]

To elucidate the potential downstream pathways through which FGF or HRG signals may operate during SC‐astrocyte mixing, we treated boundary assays of the various glial cell types with chemical inhibitors of the MAP kinase, PI3 kinase, and AKT signaling pathways. MAPK and PI3/AKT cascades are the two main transduction routes downstream of receptor tyrosine kinases, such as FGF or erbB receptors. SC migration and proliferation have been previously linked to MAPK activity (Kim et al., 1997; Meintanis et al., 2001), however in the presence of the MAP kinase inhibitor PD98059, formation of SC‐astrocyte boundaries was unchanged (Fig. 4A,B). Similarly the interaction of SC‐S1S2 and OECs with astrocytes were unaffected by PD98059 treatment (Fig. 4A,B).

PI3K/AKT signaling is important for SC survival and myelination (Campana et al., 1999; Li et al., 2001, 2004). In contrast to MAPK blockade, the PI3 kinase inhibitors GSK1059615 and Wortmannin prevented FGF treated SC‐astrocyte cell mixing (Fig. 4A,B), as did the AKT inhibitor (Fig. 4A,B). SC‐S1S2‐astrocyte, and OEC‐astrocyte cell mixing were also strongly blocked by PI3K or AKT inhibitor treatments (Fig. 4A,B). These data suggest that the PI3K‐AKT signaling pathway is important in the breakdown of SC‐astrocyte boundaries mediated by FGF or S1S2 treatments, and may also be active during OEC‐astrocyte intermingling.

### Integrins Are Important Mediators of Cell Mixing in Boundary Assays

**Figure 5:**
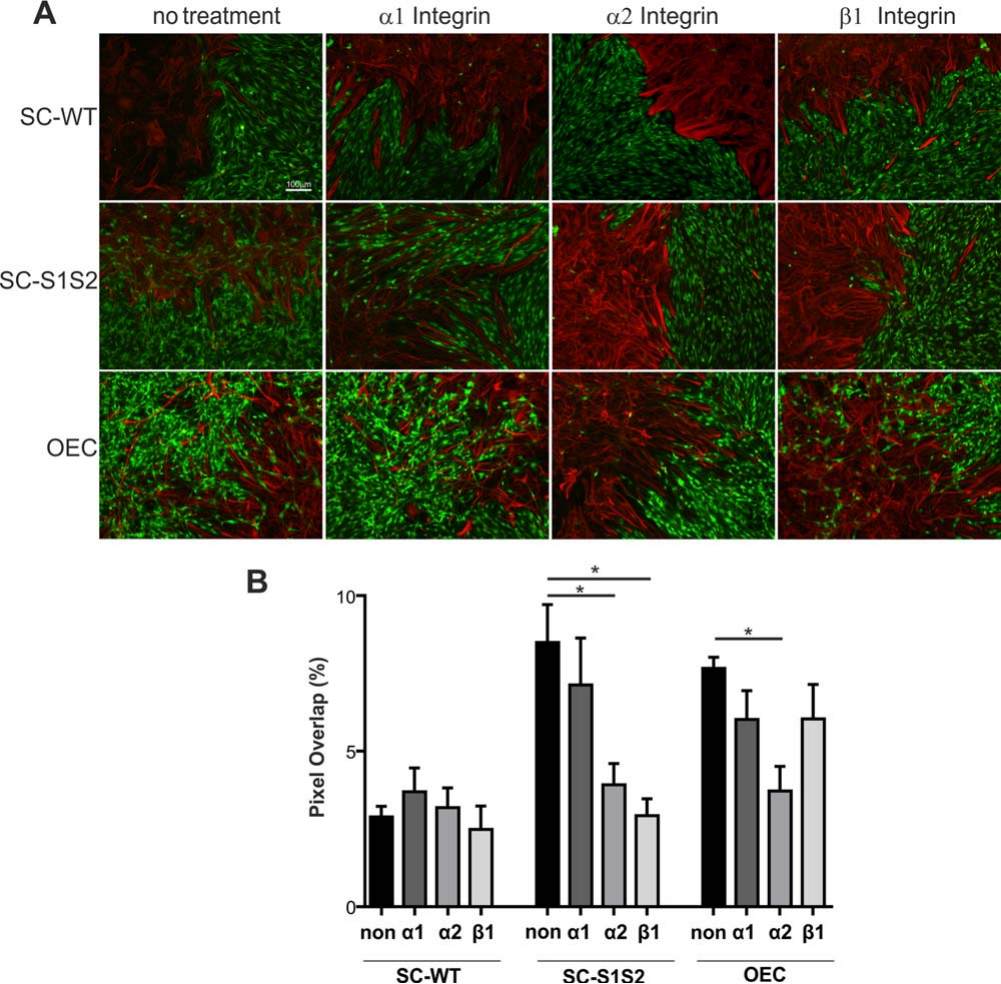
Integrins are required for sulfatase‐mediated Schwann cell motility. **A**: Control SC‐WT‐astrocyte boundaries are not affected by antibody mediated inhibition of alpha1, alpha2, or beta1 integrin function. SC‐S1S2‐astrocyte cell mixing is reduced in the presence of alpha2 or beta1 integrin function‐blocking antibodies, alpha1 integrin antibodies had no effect. OEC‐astrocyte cell mixing was blocked in the presence of alpha2 integrin function blocking antibodies only. **B**: Quantification of boundary assays treated with integrin function blocking antibodies: alpha2 antibodies significantly reduce SC‐S1S2‐astrocyte (*n* = 4; *P* = 0.047) and OEC‐astrocyte (*n* = 5; *P* = 0.011) cell mixing, beta1 antibodies reduce SC1S2‐astrocyte cell mixing (*n* = 3; *P* = 0.027). Scale bar represents 100 μm in all images. [Color figure can be viewed at wileyonlinelibrary.com]

Integrins have been implicated in promoting SC migration across inhibitory aggrecan substrates secreted by astrocytes via PI3K/AKT activation (Afshari et al., 201). We therefore tested whether integrins were similarly involved in the breakdown of glia‐astrocyte boundaries using integrin function‐blocking antibodies. Integrin blockade had no effect on control SC‐WT‐astrocyte boundary assays (Fig. 5A,B), however alpha2 and beta1 integrin function‐blocking antibodies significantly reduced SC‐S1S2‐astrocyte mixing (Fig. 5A,B). OEC‐astrocyte cell mixing was also decreased in the presence of alpha2 integrin function‐blocking antibodies, although alpha1 and beta1 treatments had no effect (Fig. 5A,B).

Overall, these in vitro data support the hypothesis that SCs engineered to express sulfatases display increased integrin‐dependent motility via modulation of NRG and FGF receptor‐linked PI3K/AKT intracellular signaling.

### Sulfatase Reduces Astrocyte Reactivity in Vivo

**Figure 6:**
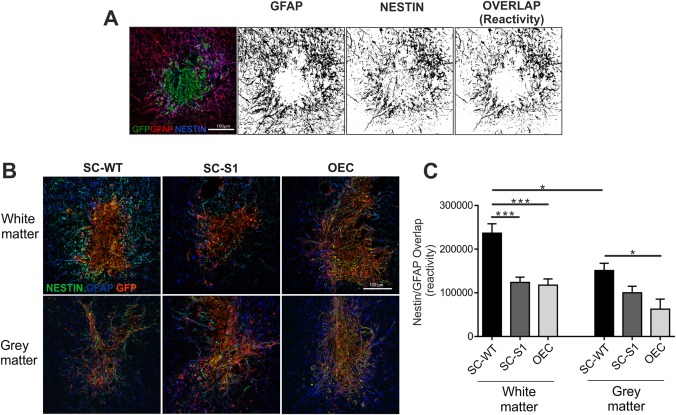
SC engineered to express sulfates reduces local astrocytic reactivity in spinal cord white matter. **A**: Levels of astrocytic reactivity around injection sites were estimated by immunostaining for GFAP and nestin. Pixel overlap of GFAP/nestin was by splitting original TIFFs into red and green channels, creating black and white binary images, then counting overlapping pixels. The combination of GFAP and nestin provided a more accurate indication of local reactivity than either marker alone. **B,C**: SC‐WT, SC‐S1, or OECs were injected into normal (uninjured) spinal cord white matter or gray matter. Astrocyte reactivity was measured by immunostaining for GFAP (blue) and nestin (green). Confocal projections of injury sites analyzed for overlap of green and blue pixels using ImageJ. Control SC‐WT injections into white matter resulted in significantly more glial reactivity than SC‐S1 injections (*n* = 8; *P* = 0.0002), or OEC injections (*n* = 7; *P* = 0.0001) into white matter. SC‐WT injections into white matter also induced more astrogliosis than SC‐WT injections into grey matter (*n* = 8; *P* = 0.0021). OEC injections into grey matter induced less astrocyte reactivity than SC injections into gray matter (*n* = 4; *P* = 0.0164), although SC‐S1 were not significantly different from SC = wt following gray matter injections. Scale bar represents 100 μm in all images. [Color figure can be viewed at wileyonlinelibrary.com]

A crucial aspect of cell transplantation into the injured spinal cord is the extent to which the transplanted cells themselves may cause an aggressive astrocytic response. Different cells types are known to promote different reactions: injections of fibroblasts or olfactory mucosa into normal spinal cord promote a more extensive upregulation of GFAP than injections of SCs or OECs for example (Toft et al., 2013). After injection of cells into the white matter we observed that SC‐S1 resulted in significantly reduced levels of astrocyte reactivity compared to control SC‐WT (Fig. 6). We measured the local astrocyte reactivity surrounding each injection site by immunostaining for nestin (an intermediate filament upregulated in reactive astrocytes; Eliasson et al., 1999) and GFAP and calculating the degree of overlap in immunoreactivity. This method effectively removed normal background levels of GFAP‐IR from our measurements allowing assessment of injected cell induced changes (Fig. 6A). The introduction of sulfatase into SC‐WT reduced astrocyte reactivity to approximately the same level as for OECs, which have been shown to promote less astrogliosis than normal SCs in spinal cord white matter (Lakatos et al., 2003). Interestingly, SC‐WT injections into grey matter (dorsal horns) led to less reactivity than comparable injections into white matter (Fig. 6B). As seen in white matter, OECs injected into grey matter produced less nestin/GFAP‐IR upregulation than SC‐WT in grey matter, although SC infected with Sulf did not result in a significant reduction in SC reactivity (Fig. 6A,B). Injections of SC‐S1S2 into the normal cord did not produce any different effects on GFAP/nestin‐IR when compared to SC‐WT cells (data not shown).

## Discussion

We have previously shown that OECs express higher levels of sulfatases than SCs; leading to the hypothesis that differential HSPG sulfation may underpin the different behaviors of OECs and SCs in the presence of astrocytes. Supporting functional evidence came from RNAi experiments showing that knockdown of OEC sulfatases promoted the formation of boundaries with astrocytes (Higginson et al., 2012). The results described here strengthen the support for the hypothesis, showing that SCs engineered to express sulfatases mix with astrocytes more than untreated SCs and can be considered to be more OEC‐like in character.

**Figure 7:**
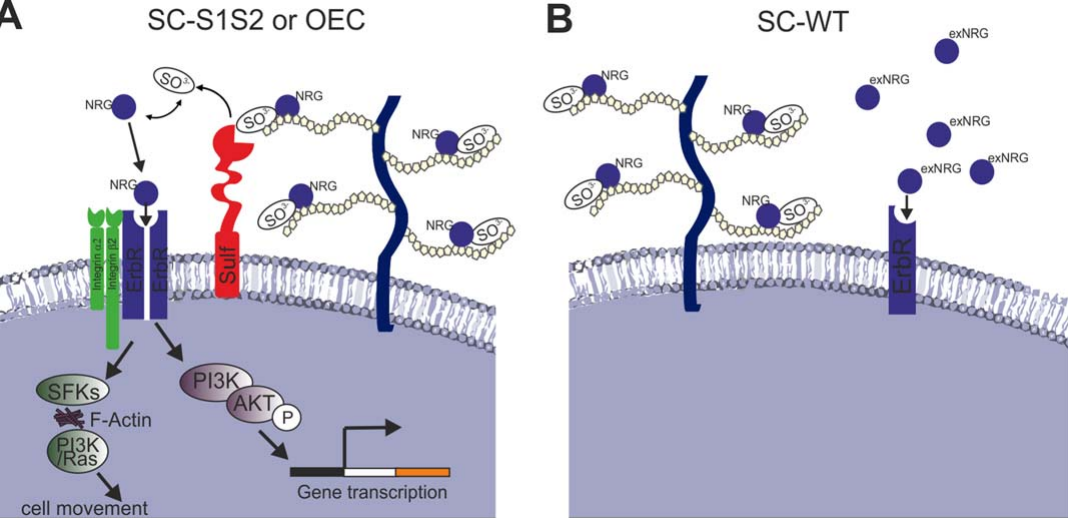
Schematic illustrating possible mechanism for OECs and Sulf1/2 expressing SCs to mingle with astrocytes. **A**: The glial cells expressing sulfatase on their cell surface can remove the heparin binding NRG from the HSPG in the ECM allowing the activation of the erbB receptor leading to PI3K/AKT activation and movement of the cell into the astrocyte environment. **B**: Since SC‐WT do not express sulfatase they cannot release NRG into the environment and therefore stay in a boundary with astrocytes; however, on the application of high levels of exogenous NRG (exNRG) SC‐WT can be forced to mingle. The activity is dependent on integrins but not MAPK. Integrins have been reported to associate with the erbB receptors which activates src family kinases (SFKs), e.g. fyn to activated cell movement via PI3K/Ras. [Color figure can be viewed at wileyonlinelibrary.com]

It is well documented that the growth and motility of SCs is regulated by neuregulins (NRGs/HRG) in particular NRG1 (Mahanthappa et al., 1996; Meintanis et al., 2001) with an additional requirement for high intracellular cAMP levels (Raff et al., 1978). During development, SCs require axonally derived NRG1 for survival; this helps maintain the one‐to‐one relationship between axons and SCs in the peripheral nervous system. Additionally, NRG1 is released by astrocytes following CNS injury (Tokita et al., 2001), and secreted by cultured astrocytes into the media (Pollock et al., 1999), so the astrocytes themselves are a likely NRG source. Interestingly, it has been shown that NRGs bind to cell surface HSPGs present on SCs and this interaction is essential for its activity (Sudhalter et al., 1996). Indeed, removal of cell surface HSPGs by inhibition of proteoglycan biosynthesis or heparitinase treatment blocks NRG‐mediated SC proliferation (Ratner et al., 1985). However, high levels of HSPG can act to inhibit NRG signaling, while the addition of exogenous heparin or HS, sequesters soluble NRG in culture and blocks SC proliferation (Sudhalter et al., 1996). In our experiments, we propose that tranduction with sulfatases may alter NRG‐HSPG binding, leading to potentiation of NRG activity by release of ligand from extracellular reservoirs. The sulfation profile of HSPG is an important determinant of NRG‐HSPG interactivity, since it has been shown that removal of N‐sulfate, 2‐O‐sulfate, or 6‐O‐sulfate groups results in reduced NRG1‐binding (Pankonin et al., 2005). It is possible that sulfatase, acting on soluble or membrane‐associated HSPGs in boundary assays, reduces the affinity of NRG‐HSPG binding, freeing the ligand to activate erbB receptors on the SC surface (see schematic in Fig. 7). A similar model has been proposed to describe the effects of sulfatase on Wnt signaling. It was proposed that highly sulphated HSPGs sequester Wnt ligand in the ECM, while sulfatase activity reduces the affinity of Wnt‐HSPG binding thus increasing Wnt bioavailability and receptor activation (Ai et al., 2006). Moreover, it is known that SC migration in scratch assays is predominantly mediated by MAP Kinase signaling downstream of NRG‐erbB, although PI3K is also important to a lesser extent (Meintanis et al., 2001). Furthermore, PI3K is activated downstream of NRG‐erbB interaction during SC survival (Li et al., 2001). Our findings that PI3K blockade prevented sulfatase‐mediated SC motility may therefore reflect an inhibition of NRG signaling. Moreover, we demonstrate that the concentration of NRG/HRG can have differential biological effects on glia cells.

In contrast to our previous confrontation assay studies (Lakatos et al., 2000), we report here that FGF signaling promotes SC‐astrocyte mixing in vitro; however, this may reflect the modifications of the assay media to contain HRG (for better survival of Schwann cells) and experimental timing (e.g., shorter contact time before cell meeting). Although some studies have suggested that FGF is mitogenic for SCs (Ratner et al., 1985), particularly in the presence of serum (Watabe et al., 1994), others have reported that FGF has no effect (Raff et al., 1978) or is dependent on the presence of forskolin (Davis and Stroobant, 1990). Our assays are conducted in the presence of forskolin, so FGF‐induced SC proliferation may be occurring, although this is not required for NRG‐induced cell migration (Meintanis et al., 2001). Astrocytes are also influenced by FGFs as cultured astrocytes express FGF receptors (Reilly et al., 1998), and FGF is known to promote astrocyte survival, proliferation and migration (Hou et al., 1995; Petroski et al., 1991). Following CNS injury, astrocyte FGF2 is upregulated (Logan et al., 1992) and acts in an autocrine manner to further increase levels of FGF2 and FGF receptor (Gomez‐Pinilla et al., 1995). Astrocytes also secrete HSPGs (Johnson‐Green et al., 1991), and HS can modulate FGF‐dependent astrocyte proliferation (Gómez‐Pinilla et al., 1995, 1996). Furthermore, HS levels are upregulated in response to CNS injury (Leadbetter et al., 2005). It has been demonstrated that sulfatases modulate this response by limiting HSPG‐FGFR interaction and thus inhibit FGF signaling (Otsuki et al., 2010; Wang et al., 2004). We can therefore propose a model in which our transplanted sulfatase‐expressing SCs influence the local ECM, reducing the 6‐O‐sulfation levels of HSPG, and preventing formation of FGF‐HSPG‐FGFR ternary complexes. This blockade of FGF signaling would be predicted to reduce levels of astrocytic proliferation and reactivity.

It is possible therefore that FGF‐dependent SC‐astrocyte mixing may occur mainly by direct astrocytic effects, however the regulation of this property is complex and it may be regulated not just by FGF and HRG but also Eph/eprin (Afshari et al., 2010).

Our results also confirm that integrins play an important role during SC‐astrocyte mixing. We show that alpha2 and beta1 function‐blocking integrin antibodies block SC‐S1S2‐astrocyte intermingling, suggesting that integrins regulate the SC‐astrocyte interface and are affected by alterations in extracellular HSPG sulfation. These finding are in agreement with Afshari et al. (2010) who demonstrated that inhibition of integrin function by the CSPG aggrecan reduces SC motility on astrocytes, while integrin activation allows SCs to overcome inhibitory astrocyte substrates. Beta1 integrin modulates SC migration on laminin substrates (Milner et al., 1997), suggesting that laminin may be important during cell mingling. Further evidence comes from experiments in which heparin treatment blocked laminin‐dependent SC spreading in culture; this effect was synergistic with anti‐integrin antibody treatment (Carey et al., 1990). Although the major role of integrins has been thought to be in mediating the adhesion between cells and the ECM increasing evidence suggest that integrins can regulate signaling pathways (Soung et al., 2010). Thus, it is also possible that integrins may play a more direct role in controlling SC movement; integrins are directly involved in NRG‐signaling in some cancer cells, and influencing NRG‐erbB signals (Ieguchi et al., 2010). Furthermore, HSPGs and heparin can directly bind to integrin molecules to influence cell behavior (Battaglia et al., 1993; Faye et al., 2009). It has been suggested that integrin‐ECM interaction can significantly amplify growth factor‐meditated signaling events (Somanath et al., 2007) and that there is crosstalk between integrin and the erbB receptor which then activates fyn leading to cell movement via F‐actin and PI3K/Ras (Soung et al., 2010 see Fig. 7). Thus, we speculate that sulfatase activity may also affect HSPG‐integrin binding and thus could influence integrin signaling in a direct manner.

Our data have therefore demonstrated an increase in SC motility in vitro when engineered to overexpress sulfatase, overcoming their default tendency to form boundaries with astrocytes. De‐sulfation of extracellular HSPGs by sulfatases is likely to lower the binding affinity of HRG to ECM HSPGs, resulting in release of HRG ligand and activation of erbB receptors. PI3K/AKT‐mediated transduction of the signal then directs cell movement via integrin‐dependent mechanisms (Fig. 7).

To assess if the level of sulfatase secreted by the cells would influence astrocyte reactivity in vivo we injected the various glial cell types into white matter of the normal spinal cord. Our data demonstrated that there was less upregulation of nestin and GFAP, markers of reactivity, in sulfatase expressing SCs than control SC‐WT. Moreover, astrocyte reactivity surrounding the SC injection site was reduced to levels similar to that of OEC injections. Interestingly, when the same panel of cells were injected in the grey matter of the normal spinal cord we noted a previously undescribed difference in the response of white and grey matter of the cells. We found there was no difference in reactivity following injection of sulfatase‐expressing SCs and SC‐WT into grey matter even though there was still a difference in reactivity when compared to OEC injections. This may be due to the lower levels of reactivity seen in grey matter in general, meaning that differences between cell populations are more difficult to conclusively demonstrate. Moreover, the differences seen in response to cell injections between the white and grey matter may reflect regional differences between the astrocytes in the two tissues (Shannon et al., 2007). Other reports have shown that white and grey matter differ in their remyelinating capacity after demyelination induced by cuprizone, this effect was proposed to be due to differences in microglial infiltration (Gudi et al., 2009). It is possible that the regional differences in astroglia reactivity described here may reflect similar differences in microglial infiltration.

The species orthologs of the Sulfs are highly conserved with human and murine proteins showing 93‐94% amino acid identity and S1 and S2 are 63–65% identical within the same species (Morimoto‐Tomita et al., 2002). It has been suggested that there may be some functional redundancy between S1 and S2 although others have suggested that they have different functions and can differentially regulate HS sulfation (Kalus et al., 2015, 2009). Our data suggest both Sulf1 and Sulf2 expression in SCs was required for cell mingling in vitro but not for the astrocyte response in vivo. This could simply reflect that an increased level of sulfatase activity in the double infected cells is necessary for affecting function in vitro but works via a different mechanism when interacting with astrocytes in vivo. This is possible as comparative studies on the activity of Sulfs in vitro and in vivo show that there was a highly restricted 6‐0‐sulfate substrate specificity for the Sulfs in vitro which was in contrast to more dynamic effects of Sulf loss on N‐, 2‐O‐, and 6‐O‐sulfated moieties in vivo. Thus the Sulfs may well behave differently when in the more complex environment of the ECM (Dai et al., 2005; Ai et al., 2006; Lamanna et al., 2008; Kalus et al., 2015). It is apparent that Sulf1 and Sulf2 function is complex as seen from studies by Lammana et al. who generated single and double knock‐out mice for the two murine endosulfatases mSulf1 and mSulf2. Detailed structural analysis of HS from fibroblast of the msulf −/− KO mice showed that they had a significantly higher increase in 6‐O‐sulfation, which was not seen in the msulf2−/− KO mice fibroblasts. Even more interesting was seen from data on the level of 6‐O‐sulfation in the double mSulf1−/−/2−/− HS showed it to be significantly higher than that observed in the mSulf1−/− counterpart. These data suggest that mSulf1 and mSulf2 are functionally co‐operative and that, although increased mSulf1 expression can compensate for loss of mSulf2 activity, mSulf2 is unable to fulfill the role of mSulf1 (Lamanna et al., 2006). It has also been shown that there is a highly restricted 6‐O‐sulfate substrate specificity for the Sulfs in vitro, which was contrasted by dynamic effects of Sulf loss on N‐, 2‐O‐, and 6‐O‐sulfated moieties in vivo (Lamanna et al., 2008).

Overall, our data demonstrate that specific enzymatic modification of the HS content of the ECM can affect 2 distinct signaling pathways in ways potentially beneficial for their interaction with astrocytes. Firstly, SCs expressing sulfatase are able to overcome astrocytic barriers in vitro, a possible correlative prerequisite for effective integration into the CNS. Second, local astrogliosis is reduced, possibly due to inhibition of FGF‐FGFR signaling (Kang et al., 2014)

The use of SCs modified to secrete sulfatases may therefore provide a strategy for minimizing astrocytic reactivity and proliferation around a transplanted injury site. Minimizing the molecular and physical barriers associated with reactive astrocytes should provide greater opportunity for axonal sprouting and regeneration and may therefore enhance the effectiveness of SC transplantation as a therapy for spinal cord repair. Collectively these effects have the potential for exploitation in the development of novel therapeutic strategies for CNS repair.
